# A Case of Postural Orthostatic Tachycardia Syndrome Secondary to the Messenger RNA COVID-19 Vaccine

**DOI:** 10.7759/cureus.14837

**Published:** 2021-05-04

**Authors:** Sujana Reddy, Satvik Reddy, Manish Arora

**Affiliations:** 1 Internal Medicine, Alabama College of Osteopathic Medicine, Dothan, USA; 2 Internal Medicine, Decatur-Morgan Hospital, Decatur, USA; 3 Neuroscience, University of Alabama at Birmingham, Birmingham, USA; 4 Internal Medicine/Gastroenterology, Decatur-Morgan Hospital, Decatur, USA

**Keywords:** autoimmune pots, covid 19, dysautonomia, mrna-based vaccine

## Abstract

Postural orthostatic tachycardia syndrome (POTS) is an impaction of the autonomic nervous system initiating orthostatic tachycardia. There are numerous triggers for POTS including viruses, vaccines, and an autoimmune basis. This case report is clinically relevant to better understand the pathophysiology behind the messenger RNA (mRNA) coronavirus disease 2019 (COVID-19) vaccine and the mechanism that triggers autonomic nervous system dysfunction. Furthermore, the overall goal of this case study is to report a unique side effect associated with the novel mRNA COVID-19 vaccine. A 42-year-old male, with no prior symptoms of sinus tachycardia and presyncope episodes, is diagnosed with POTS secondary to the first dose of the mRNA COVID-19 vaccine. Symptoms to this date include sinus tachycardia, dizziness, headaches, and fatigue that are often triggered after a large meal or standing for a longer duration. Numerous diagnostic tests and images failed to confirm any other diagnosis other than POTS. There was a sequential connection between the onset of symptoms approximately one week after taking the first dose of the mRNA COVID-19 vaccine. Currently, POTS in this patient is controlled by lifestyle modification. This case report has broader implications as it can help us understand how the mRNA vaccine works on the body relative to the immune system. Our theory is that the development of antibodies activates an autoimmune reaction that triggers POTS disease. The prevalence of the POTS dysautonomia post-vaccination will be clearer as more data and research are conducted on the side effects from the innovative mRNA vaccines created to combat severe acute respiratory syndrome coronavirus 2.

## Introduction

Postural orthostatic tachycardia syndrome, POTS, is a blood circulation disorder that impacts the autonomic nervous system. POTS is characterized by significant orthostatic tachycardia via the stand test, defined as an increase in heart rate of at least 30 beats per minute, without remarkable changes in blood pressure. Patients with POTS present with the following symptoms: generalized fatigue, nausea, dizziness, heart palpitations, near syncope, light-headedness, and/or exercise intolerance; symptoms are induced by heat, food ingestion, and physical exertion [1,2]. POTS most commonly affects young female individuals between 15 and 40 years of age with a prevalence in the general population of 170 cases per 100,000 individuals [3]. 

The pathophysiology of POTS remains ambiguous. Some of the common causes of the disorder include the hypothalamus-pituitary-adrenal axis dysfunction, mitochondrial and mast cell disorders, cardiac sympathetic dysautonomia, irregular activation of the renin-angiotensin system, or a baroreflex dysfunction [3,4]. However, proposed mechanisms suggest an autoimmune basis with autoantibodies toward alpha-1 adrenergic receptors, beta-1/beta-2 receptors, M1/M2 muscarinic receptors, and ganglionic N-type acetylcholine receptors [3-5]. Onset of de novo POTS has been associated with viral infections, trauma, stress, surgery, and vaccinations. There have been studies, most notably in Denmark, that found de novo POTS after quadrivalent human papillomavirus (HPV) vaccine jabs [6,7]. The goal of this case report is to understand the pathophysiology behind how the messenger RNA (mRNA) vaccine might have triggered the autonomic nervous system inducing POTS in the patient discussed in this article.

## Case presentation

A 42-year-old male presented to the clinic with sinus tachycardia and presyncope episodes approximately six days after taking the first dose of Pfizer Bio-nTech coronavirus disease 2019 (COVID-19) vaccine. Prior to this, the patient received other vaccinations (influenza, tetanus, diphtheria, pertussis, measles, mumps, rubella, and hepatitis B) and reported no adverse side effects. Past medical history includes hypothyroidism that is well controlled with daily 125 mg of levothyroxine and B-12 deficiency that is treated with B-12 shots. There are no known medication, food, or drug allergies or anaphylactic reactions noted in history. Left-sided orchiectomy for cryptorchidism is noted in past surgical history. Patient is a social drinker but denies smoking history or illicit drug use.

Within 24 hours after taking the Pfizer COVID-19 vaccine, the patient began to experience generalized fatigue, headache, and myalgia. These symptoms persisted unabated for the next seven days. On day 7, the patient developed a syncopal episode and sinus tachycardia. Since the onset of symptoms, the patient randomly exhibits near-fainting episodes when standing for a long time, intermittent palpitations, anxiety, sleep disturbances, and occasional numbness in the lower extremities. The second dose of the Pfizer COVID-19 vaccine was not given due to the severity of the symptoms mentioned.

Multiple physical examinations, extensive diagnostic tests, and laboratory tests have failed to provide a reliable explanation for the patient’s clinical symptoms. Polymerase chain reactions for severe acute respiratory syndrome coronavirus 2 (SARS-CoV2) and SARS-CoV2 antibodies were negative. Complete blood count, basic metabolic panel, and comprehensive metabolic panel were within normal limits. Cholesterol panel did indicate elevated total cholesterol with a value of 225 (normal 0-200 mg/dL) and low-density lipoprotein of 152 mg/dL (<100 mg/dL). Other special chemistry tests, such as 8 am cortisol levels, C reactive protein, troponin T/I, CK profile, ferritin, and pro-B-natriuretic peptide, were within normal limits. To rule out pheochromocytoma or other neuroendocrine tumors, a 24-hour urine analysis for vanillylmandelic acid test was done and was negative for any metanephrines. Ultrasound of abdomen was essentially unremarkable with a 1.9 cm simple left renal cyst noted. Ultrasound of the renal artery was performed to rule out essential hypertension, and the findings noted no doppler evidence of hemodynamically significant renal artery stenosis. Carotid doppler was also negative. Head CT scan did not show any findings. Echocardiogram was conducted and showed no diastolic or systolic dysfunction with normal valvular structures and an ejection fraction of 71%. During the echocardiogram study, patient had sinus tachycardia that were clocked at 158 beats per minute and spontaneously lowered to 126 beats per minute. Per cardiology, a 31-day Holter monitor was placed to monitor the heart rhythm and showed episodes of sinus tachycardia. Patient continues to exhibit symptoms and is currently treated with lifestyle modifications, such as wearing compression socks and increased sodium intake.

## Discussion

A healthy patient with persistent unexplained symptoms, such as chronic fatigue, presyncope, and inappropriate sinus tachycardia, is often misdiagnosed because of the poorly understood pathophysiology and etiologies for dysautonomia diseases. POTS is often delayed during diagnosis due to overlap with other dysautonomia diseases, such as inappropriate tachycardia syndrome or anxiety-related disorders [8,9]. Research indicates that POTS is an exaggerated response of orthostatic tachycardia. For example, when a normal healthy patient moves to the upright position, gravity pools blood downward. The brain senses this change, causing the blood vessels in the lower extremities to constrict forcing blood to return to the heart for circulation. In contrast, POTS patients are unable to maintain or make blood vessel constriction. Therefore, the brain compensates by increasing heart rate, which is a landmark sign for dysautonomia [1,2,9]. 

It is postulated that disruption of the body’s autonomic nervous system in POTS has potential triggers from viral infections and vaccines. De novo POTS is noted in a number of cases post-vaccination of HPV. One case report noted that there is a possible temporal relationship between the vaccination of Gardasil and onset of POTS [7,10]. Numerous post-vaccination disorders, such as Guillain-Barre syndrome and transverse myelitis, have been reported to exhibit cross-reacting antibodies. These antibodies are proposed to mechanistically react beyond target ganglionic acetylcholine receptors, and thus could explain the POTS pathogenesis in the setting of post-vaccination [11]. One study examined 433 patients in which 155 patients were diagnosed with POTS after vaccination. This study proposed that a possible explanation for the increase of dysautonomia syndromes could be through an autoimmune mechanism by way of autoantibodies [12]. 

The autoimmune pathophysiology might be a key component to understand POTS and has been studied in many reports. One study emphasized that POTS patients manifest autoantibodies to the adrenergic receptors, specifically the α1‐adrenergic receptor, that impair the vasoconstrictor response. The autoantibodies are thought to block normal ligand binding of norepinephrine to the α1‐adrenergic receptor thus impeding the homeostatic regulation of autonomic nervous system function leading to an increase in sympathetic nervous activity and baroreceptor activation [4,5,11,12]. This increased response leads to tachycardia. Another study conducted at the Mayo Clinic found organ-specific autoantibodies in POTS patients; furthermore, their results support that about 33% of POTS cases have an autoimmune basis. This study also suggested that one major cause of POTS is autoantibodies targeting cardiac and vascular adrenergic receptors [13]. Finally, another study suggests that POTS can result from a disruption in angiotensin-converting enzyme 2 (ACE2) receptor dysfunction [14]. The mechanism for the ACE2 defect in POTS is poorly understood but could be genetically linked. This leads us to SARS-CoV-2 as this virus gains entry by attaching to the ACE2 receptor.

Viral infections, such as human immunodeficiency virus, hepatitis C, mumps, Epstein-Barr virus, and influenza, have been reported with acute dysautonomia. A case study recently reported dysautonomia secondary in a critically-ill SARS-CoV-2 patient. This study explained how there was damage to the afferent baroreceptor pathway, from the carotid bodies up to the nucleus tractus solitarius, leading to autonomic nervous system dysfunction [15]. Furthermore, it is possible that the anti-SARS-CoV2 antibodies might have an autoimmune basis as they cross-react with the receptors in the ganglia [16]. Another case study found POTS in a SARS-CoV2 patient hypothesized that the virus reduces the ACE2 receptor when it binds and consequently increased sympathetic activity [17]. Given the nature of the aforementioned study findings, it is important to look at how COVID-19 and POTS are related. This leads us to understand the COVID-19 vaccine and how the mechanism of the mRNA vaccine might trigger a similar response to autoantibodies.

 Pfizer BioNTech COVID-19 vaccine utilizes a nucleoside-modified mRNA, from the genetic code of the virus, that is translated in the host’s cells to make a spike protein target. This spike protein acts as an intracellular antigen, which in turn stimulates the immune system to form neutralizing antibodies that will block viral entry into host cells [18]. It is our speculation that the formation of antibodies triggers an autoimmune response that stimulates POTS disease, like it does in the patient in this case. To date, there is no published case report of POTS disease secondary to an mRNA vaccine. Since this is a new vaccine, more research and data collection on side-effect profiles will need to be done to see how prevalent this dysautonomia is post-vaccination.

## Conclusions

POTS is a syndrome that is difficult to diagnose and often debilitating for the patient. As with the patient discussed in this case, there was a temporal association between the onset of symptoms and the first dose of the mRNA COVID-19 vaccination. Prior to this event, the patient had no significant symptoms, and, therefore, it is our hypothesis that the reason for the patient’s onset of POTS was likely associated with the mRNA vaccine. This is the first case published to date reporting this side-effect profile; there still needs to be more research done on adverse effects due to the new mRNA vaccines. 

## References

[1] Thieben MJ, Sandroni P, Sletten DM (2007). Postural orthostatic tachycardia syndrome: the Mayo clinic experience. Mayo Clin Proc.

[2] Fu Q, Vangundy TB, Galbreath MM (2010). Cardiac origins of the postural orthostatic tachycardia syndrome. J Am Coll Cardiol.

[3] Mathias CJ, Low DA, Iodice V, Owens AP, Kirbis M, Grahame R (2011). Postural tachycardia syndrome--current experience and concepts. Nat Rev Neurol.

[4] Li H, Yu X, Liles C (2014). Autoimmune basis for postural tachycardia syndrome. J Am Heart Assoc.

[5] Dubey D, Hopkins S, Vernino S (2016). M1 and M2 muscarinic receptor antibodies among patients with postural orthostatic tachycardia syndrome: potential disease biomarker. J Clin Neuromuscul Dis.

[6] Brinth L, Theibel AC, Pors K, Mehlsen J (2015). Suspected side effects to the quadrivalent human papilloma vaccine. Danish Med J.

[7] Blitshteyn S (2014). Postural tachycardia syndrome following human papillomavirus vaccination. Eur J Neurol.

[8] Bryarly M, Phillips LT, Fu Q, Vernino S, Levine BD (2019). Postural orthostatic tachycardia syndrome: JACC Focus Seminar. J Am Coll Cardiol.

[9] Benarroch EE (2012). Postural tachycardia syndrome: a heterogeneous and multifactorial disorder. Mayo Clin Proc.

[10] Raj SR (2006). The postural tachycardia syndrome (POTS): pathophysiology, diagnosis & management. Indian Pacing Electrophysiol J.

[11] Fedorowski A (2019). Postural orthostatic tachycardia syndrome: clinical presentation, aetiology and management. J Intern Med.

[12] Hviid A, Thorsen NM, Valentiner-Branth P, Frisch M, Mølbak K (2020). Association between quadrivalent human papillomavirus vaccination and selected syndromes with autonomic dysfunction in Danish females: population based, self-controlled, case series analysis. BMJ.

[13] Singer W, Klein C, Low P, Lennon V (2015). Autoantibodies in the postural tachycardia syndrome (P1.272). Neurology.

[14] Mustafa HI, Raj SR, Diedrich A (2012). Altered systemic hemodynamic and baroreflex response to angiotensin II in postural tachycardia syndrome. Circ Arrhythm Electrophysiol.

[15] Eshak N, Abdelnabi M, Ball S, Elgwairi E, Creed K, Test V, Nugent K (2020). Dysautonomia: an overlooked neurological manifestation in a critically ill COVID-19 patient. Am J Med Sci.

[16] Kanjwal K, Jamal S, Kichloo A, Grubb BP (2020). New-onset postural orthostatic tachycardia syndrome following coronavirus disease 2019 infection. J Innov Card Rhythm Manag.

[17] Umapathi T, Poh MQW, Fan BE, Li KFC, George J, Tan JYL (2020). Acute hyperhidrosis and postural tachycardia in a COVID-19 patient. Clin Auton Res.

[18] Polack FP, Thomas SJ, Kitchin N (2020). Safety and efficacy of the BNT162b2 mRNA Covid-19 vaccine. N Engl J Med.

